# Precipitation increase counteracts warming effects on plant and soil C:N:P stoichiometry in an alpine meadow

**DOI:** 10.3389/fpls.2022.1044173

**Published:** 2022-11-02

**Authors:** Lina Shi, Zhenrong Lin, Xiaoting Wei, Cuoji Peng, Zeying Yao, Bing Han, Qing Xiao, Huakun Zhou, Yanfang Deng, Kesi Liu, Xinqing Shao

**Affiliations:** ^1^ College of Grassland Science and Technology, China Agricultural University, Beijing, China; ^2^ Institute of Ecological Protection and Restoration, Chinese Academy of Forestry, Beijing, China; ^3^ College of Grassland Science, Gansu Agricultural University, Lanzhou, China; ^4^ Key Laboratory of Restoration Ecology of Cold Area in Qinghai Province, Northwest Institute of Plateau Biology, Chinese Academy of Science, Xining, China; ^5^ Qilian Mountain National Park Qinghai Service Guarantee Center, Xining, China

**Keywords:** ecological stoichiometry, warming, precipitation increase, interactive effect, alpine meadow

## Abstract

Temperature and precipitation are expected to increase in the forthcoming decades in the northeastern Qinghai-Tibetan Plateau, with uncertain effects of their interaction on plant and soil carbon:nitrogen:phosphorus (C:N:P) stoichiometry in alpine ecosystems. A two-year field experiment was conducted to examine the effects of warming, precipitation increase, and their interaction on soil and plant C:N:P stoichiometry at functional groups and community level in an alpine meadow. Warming increased aboveground biomass of legumes and N:P ratios of grasses and community, but did not affect soil C:N:P stoichiometry. The piecewise structural equation model (SEM) indicated that the positive effect of warming on community N:P ratio was mainly resulted from its positive influence on the aboveground biomass of functional groups. Precipitation increase reduced C:N ratios of soil, grasses, and community, indicating the alleviation in soil N-limitation and the reduction in N use efficiency of plant. SEM also demonstrated the decisive role of grasses C:N:P stoichiometry on the response of community C:N:P stoichiometry to precipitation increase. The interaction of warming and precipitation increase did not alter plant community and soil, N:P and C:P ratios, which was resulting from their antagonistic effects. The stable soil and plant community C:N:P stoichiometry raised important implications that the effect of warming was offset by precipitation increase. Our study highlights the importance of considering the interaction between warming and precipitation increase when predicting the impacts of climate change on biogeochemical cycles in alpine meadow ecosystems.

## Introduction

In recent decades, ongoing global climate changes (e.g., warming, altered precipitation, elevated carbon dioxide, and nitrogen (N) deposition), induced by human activities such as fossil fuel combustion and fertilizer application (IPCC, 2017; Bai et al., 2020), have caused prominent effects on terrestrial ecosystems, especially on the sensitive and fragile grassland ecosystems at high altitudes (Chen et al., 2013). The Qinghai-Tibetan Plateau (QTP), the Eurasian continent’s largest geo-morphological unit (Song et al., 2021), covers an area of 2.5 million ha at an average altitude of 4000 m above sea level (Qiu, 2008). The alpine meadow is one of the most important grassland ecosystems and accounts for 35% of the plateau area (Cao et al., 2004). In the past 50 years, the air temperature of the QTP increases at a rate of 0.4°C per decade, a pace of nearly doubling warming speed of global warming (Gai et al., 2009; Liu et al., 2018). Precipitation changes on the QTP show spatial variation, with an increasing trend in the northern and southern regions but a decreasing trend in the central regions (Wu et al., 2007). Annual precipitation is expected to increase by more than 10% by the end of the century in the northeastern QTP (Ding et al., 2007). Thus, it might be inevitable that warming and precipitation increase would occur simultaneously in the northeastern QTP in future scenarios.

Climate change may profoundly impact primary productivity of grassland ecosystems as well as change biogeochemical cycles among key elements such as carbon (C), N, and phosphorus (P) (Yue et al., 2017). C, N, and P are the key elements that form plant tissue structure and maintain plant growth and development and physiological metabolism (Chen and Chen, 2021). Ecological stoichiometry has often been employed to shed light on the relationship and feedback between soil and plants in grassland ecosystems (Bai et al., 2012), which plays an important role in driving crucial ecological progress such as plant biomass, plant community structure, and biogeochemical cycles (He et al., 2006; Dijkstra et al., 2012; Li et al., 2021b). The C:N:P ratios in environments and organisms were used as measurement criteria to track the distribution of elements among the components of ecosystems (Zechmeister-Boltenstern et al., 2015; Chen and Chen, 2021). Temperature and precipitation are key driver factors of ecosystem processes (Wu et al., 2011). Given such importance, a better understanding of plant and soil C:N:P stoichiometry in an alpine meadow response to warming and altered precipitation is critical to accurately predict changes in the interaction between plant and soil under global change scenarios.

Warming affects plant physiology and stoichiometry by directly increasing microbial activity or directly inhibiting nutrient uptake due to warming-induced drought (Yuan and Chen, 2015; Yan et al., 2022). Yue et al. (2017) found that soil showed high stoichiometric homeostasis to global warming. Elevated temperature can promote soil nitrification and net N mineralization rate (Melillo et al., 2011), but decrease the soil available P content (Dijkstra et al., 2012), leading to the positive effect on plant nutrient uptake, higher plant N:P ratio, and lower C:N and C:P ratios. Conversely, drought induced by warming can depress N and P uptake by plant (Ma et al., 2015), decreasing N and P concentration in plant tissues, and ultimately leading to a lower C:N and C:P ratios in plant (Viciedo et al., 2019). Previous studies have reported inconsistent results about the response of soil and plant stoichiometry to warming in QTP. For instance, it is generally agreed that warming increased the plant C:N ratio (Yang et al., 2011; Wang et al., 2021) and had no effect on soil C, N, P or C:N:P stoichiometry (Wang et al., 2014; Chen et al., 2021; Xu et al., 2022), but other studies reported inconsistent results (Na et al., 2011; Qin et al., 2020).

Water availability is critical to the productivity of grassland ecosystems, so precipitation is expected to be a key driver of ecosystem processes (Wu et al., 2011). Precipitation increase provides higher moisture to the soil in the plant growing area, which promotes plant growth and alters the elemental cycle in plant, roots, and soil (Sun et al., 2021). Yuan & Chen (2015) found that the increased rainfall reduced plant N:P ratio and cause a shift in the type of limiting elements for plant growth. He et al. (2006) demonstrated that there was no significant correlation between plant leaf C and P concentrations and precipitation, which implied that it was difficult to detect the response of plant C:P ratio to short-term precipitation changes. Precipitation increase promoted plant growth, resulting in more C input to the soil from litter and roots (Zhang and Xi, 2021), and promoted soil N leaching (Chen et al., 2016), leading to an increase in soil C:N ratio (Sun et al., 2021). Soil N:P ratio was greatly determined by nutrient uptake of plant (Abbasi et al., 2020). In an alpine meadow of QTP, the enhanced rainfall increased the shoot N and P content of grasses and forbs, and N:P ratio of grasses (Li et al., 2019). However, the effects of precipitation increase on soil and plant C:N:P stoichiometry were not well documented in alpine meadows.

The above findings all belong to the impacts of global change single-factor impacts on terrestrial ecosystems, while climate change scenarios were often accompanied by warming and altered precipitation. The interactive effects of warming and altered precipitation are expected to have major consequences on important ecosystem properties and processes (Wu et al., 2011; Yue et al., 2018). For instance, the increased precipitation amplified the positive impact of warming on forage quality and community biomass in alpine grasslands (Ma et al., 2017; Xu et al., 2018), but it alleviated the warming impact on plant growth in northeastern China’s Horqin sandy land (Luo et al., 2017). Furthermore, the increased precipitation offset warming effects on plant biomass and ecosystem respiration in a Tibetan alpine steppe by maintaining the balance between precipitation and evapotranspiration (Zhao et al., 2019). However, little investigation is available on the interactive effects of warming and precipitation increase on plant and soil C:N:P stoichiometry, which hinders the establishment of a sound framework for nutrient cycling in grassland ecosystems.

Temperature is one of the most important limiting factors on the QTP due to high altitude and cold climate (Zhao et al., 2018; Wang et al., 2022), and water availability is considered to the key limiting factors in controlling nutrient cycle in grassland ecosystems especially in northeastern region of QTP with low rainfall (Li et al., 2019; Yan et al., 2022). Moreover, it was reported that enhanced rainfall mitigated the water deficit induced by warming (Yu et al., 2019b; Zhao et al., 2019). To explore the effects of warming, precipitation increase, and their interaction on plant and soil C:N:P stoichiometry, we established a field experiment of simulated warming (+ 2°C) and precipitation increase (+ 20% rainfall) in an alpine meadow of the northeastern QTP and attempted to answer the following specific questions: a) Do warming and precipitation increase change plant and soil C:N:P stoichiometry? b) Does precipitation increase change the effect of warming on plant and soil C:N:P stoichiometry? We hypothesized that: a) Warming and precipitation increase change the plant and soil C:N:P stoichiometry. b) The effect of warming on plant and soil C:N:P stoichiometry is counteracted by precipitation increase.

## Materials and methods

### Site description

A field trial was conducted between 2020 and 2021 at the warming and precipitation increase simulation platform located in Haibei Autonomous Prefecture, Qinghai Province, China (36°54′59″ N, 100°56′12″ E, 3090 m above sea level, **Figure S1**). The area is typical plateau continental climate with short warm summers and long cold winters. The mean annual temperature is 1.4°C, with the maximum temperature of 27°C in July, and with the minimum temperature of -29°C in January. The mean annual precipitation is 400 mm, which mainly occurs in the plant growing season from June to August (Wei et al., 2021). The mean monthly temperature and mean monthly precipitation for 2020-2021 were shown in **Figure S2**. The annual evaporation capacity is about 1400 mm (Zhao et al., 2017). According to the Chinese soil classification system, the soil is classified as Mat-Gryic Cambisol with a clay loam texture (alpine meadow soil, Cambisols in FAO/UNESCO classification) (Ma et al., 2017). The typical vegetation type is an alpine meadow, which are dominated by species of *Elymus nutans*, *Leymus secalinus*, *Stipa purpurea*, *Poa pratensis*, *Melissilus ruthenicus*, *Kobresia humilis*, *Artemisia scoparia*, and *Potentilla chinensis*.

### Experimental design

A 30 m × 30 m meadow containing a fairly uniform mixture of plant species was fenced to eliminate the interference of livestock in 2012. The experimental facility platform was established to simulate the impacts of climate change (climate warming and precipitation increase) on the alpine meadow ecosystems in 2014. The facility platform consisted of four treatments (control (CK), warming (W, + ~ 2°C), precipitation increase (P, + 20%), and the combined treatment of warming and precipitation increase (WP)) (Wei et al., 2021). Experimental design was the randomized block design with four replicates (**Figure S1**). Each experimental plot was a circular with 2.2 m diameter and separated from the others by a 3 m buffer zone. A total of 16 circular plots were set up. All the treatments had similar topographies and land-use histories.

Eight treatments including warming were heated by open-top chambers (OTCs) with 2.2 m diameter bottom, 1.5 m diameter top, and 0.7 m height, in which the temperature was ~2°C higher than the outside. Three automatic control fans were installed on the top of each OTC to ensure that the temperature difference between the warmed and control plots is always maintained at 2°C. Eight treatments including precipitation increase were added extra water addition (20% of the precipitation) with a hand-held sprinkler after each rainfall event in the growing season (from June to August), and the amount of water added each time was calculated from the precipitation recorded by a rain gauge.

### Plant and soil sampling

We investigated the plant community characteristics (height, density, coverage) using a quadrat (1 m × 1 m) at each plot in August, 2020 and 2021, respectively. Shannon-Wiener index (H) was used to indicate the diversity of plant community. The index was calculated using the following formula (Wang et al., 2019):


$$\text{H= -}\sum_{i=1}^{\text{s}}\left({\text{P}}_{\text{i}}{\text{lnP}}_{\text{i}}\right)$$

where P_i_ is the importance values (IV) of the i-th species in all species, and S is the total number of plant species in each plot. IV of each plant species was computed as: IV = (RH + RD + RC)/3, where RH, RD, and RC are the relative height (RH), relative density (RD), and relative coverage (RC) of each plant species. The RH (or RD or RC) was equal to the height (or density or coverage) of the plant species divided by the respective sum of the heights (or densities or coverages) of all plant species in each quadrat.

The aboveground plants rather than biomass were clipped annually flush with the ground in these quadrants. According to ecological niches or functions of plant species in the grassland ecosystem, all clipped plant species were classified into four functional groups (grasses, legumes, sedges, and forbs). These plant samples were dried at 65°C for 72 h, and then weighed.

Four soil cores to depths of 0-10 cm were randomly taken from each plot using soil auger with a diameter of 3.5 cm, and mixed thoroughly to form one composite soil sample. After removing roots, litter, and stones, each composite soil sample was sieved through a 2-mm mesh. Soil samples were air-dried for physical and chemical characteristics analyses.

### Laboratory analysis

Total carbon (TC) and total nitrogen (TN) of soil and plant were measured by a C/N element analyzer (Elementar, Hanau, Germany). Total phosphorous (TP) of plant was measured by a microplate reader with ammonium molybdate and ascorbic acid as color reagents (Li et al., 2019). TP of soil was determined using the HClO_4_-H_2_SO_4_ digestion-molybdenum antimony colorimetric method (Murphy and Riley, 1962; Han et al., 2021).

We calculated community TC, TN, and TP concentrations using the following formula:


$$\text{Community TC, or TN, or TP=}\sum_{i=1}^{\text{n}}{\text{Pi}\times \text{A}}_{\text{i}}$$

where P_i_ is the proportion of the aboveground biomass of function group i to aboveground biomass of the community, and A is the TC, or TN or TP concentration of grasses, legumes, sedges, and forbs.

### Statistical analysis

The effects of warming, precipitation increase, and their interaction on plant and soil nutrients were examined using a linear mixed-effects model from the “predictmeans” package of R version 4.0.5 (The R Project for Statistical Computing, https://www.r-project.org/). Warming and precipitation increase were assigned as fixed effects, and year and plot as the random effects. Before these analyses, all data were examined for normality and log-transformed when necessary to conform with assumptions of normality and variance homogeneity. Significance levels were set at *P*<0.05.

We employed individual effects and 95% confidence intervals of effect sizes for interaction computed by Hedges’ d_+_ to classify interactive effects as significant (antagonistic or synergistic) and non-significant (additive) interactions. We regarded interactive effects as additive when the 95% CI overlaps with zero. When comparing individual effects in negative or opposite directions, their interaction effect size less than zero was regarded as synergistic and interaction effect size greater than zero as antagonistic. Finally, if the individual effects of two factors were opposite, then their sum was positive, the interaction was synergistic when it was positive, and antagonistic when it was negative, and vice versa (Hedges et al., 1999; Crain et al., 2008; Tang et al., 2019; Shi et al., 2022).

We employed piecewise structural equation models (SEM) to explore how warming, precipitation increase, and their combination affected community C:N:P stoichiometry through their effects on soil and plant characteristics. The model assumed that warming, precipitation increase, and their combination change plant community diversity, aboveground biomass of plant functional groups (extracting first component scores from principal component analysis (PCA) of aboveground biomass of grasses, legumes, sedges, and forbs), and soil C:N:P stoichiometry (extracting first component scores from PCA of soil TC, TN, TP concentration, and C:N, N:P, C:P ratios), which then alter plant functional groups C:N:P stoichiometry (extracting first component scores from PCA of TC, TN, TP concentration and C:N, N:P, C:P ratios of each plant functional group), and ultimately affect community C:N:P stoichiometry (extracting first component scores from PCA of community TC, TN, TP concentration, and C:N, N:P, C:P ratios). These were based on prior conceptual models with hypothetical relationships, which included all possible cascade pathways (**Figure S3**). The data were normalized by z-transformation and used in the analysis, incorporating random effects into the year and plot (Ma et al., 2021). We simplified the original model by sequentially removing non-significant paths until the final optimal models were acquired. The same model only contained the variables with variance inflation coefficients < 5. Directed-separation test, Fisher’s C statistic, and Akaike information criteria (AIC) were applied to evaluate the model fitness (Shipley, 2013). The SEM analyses were conducted by the “pievewiseSEM” package (Lefcheck, 2016) of R version 4.0.5.

## Results

### Soil C:N:P stoichiometry

**Figure 1:**
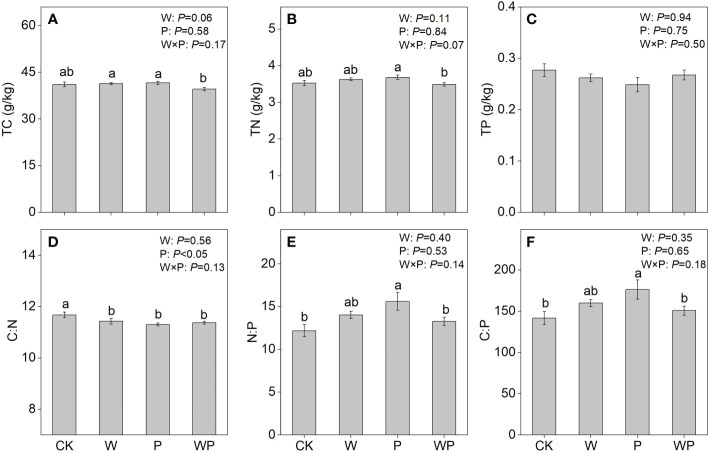
Soil total C (TC), total N (TN), total P (TP) concentration, and C:N:P stoichiometry under treatments in an alpine meadow. TC **(A)**, TN **(B)**, TP **(C)**, C:N **(D)**, N:P **(E)**, and C:P **(F)**. CK, control; W, warming; P, precipitation increase; WP, the combined warming and precipitation increase. Data are shown as means ± SE. Lowercase letters indicate differences between treatments (Duncan’s multiple comparison post hoc test: P<0.05).

**Figure 2:**
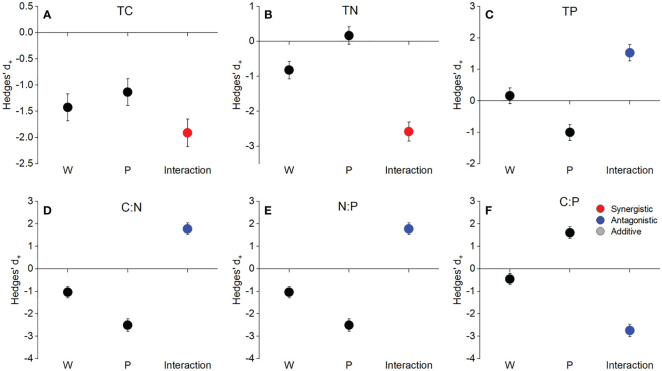
The main and interactive effects of warming and precipitation increase on soil total C, total N, total P concentration, and C:N:P stoichiometry. TC **(A)**, TN **(B)**, TP **(C)**, C:N **(D)**, N:P **(E)**, and C:P **(F)**. Values represent means with 95% confidence intervals (CIs).

Neither warming nor precipitation increase affected the soil TC, TN, TP concentrations, and N:P, C:P ratios (*P*>0.05, **Figures 1A–C, E, F**). Precipitation increase had a significant effect on soil C:N ratio (*P*<0.05, **Figure 1D**). The interaction of warming and precipitation increase had non-significant effects on soil C:N:P stoichiometry (*P*>0.05, **Figure 1**). The interactive effects of warming and precipitation increase on soil TC and TN concentrations were synergistic (**Figures 2A, B**), while antagonistic interactions were observed on soil TP concentration, C:N, N:P, and C:P ratios (**Figures 2C–F**).

### Community composition and plant aboveground biomass

**Figure 3:**
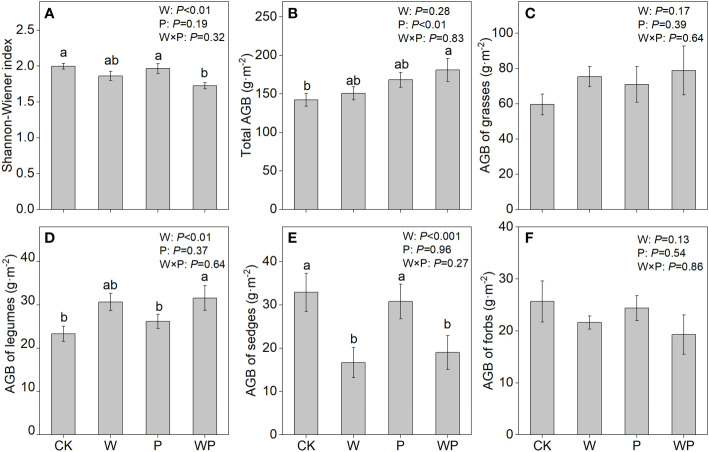
The Shannon-Wiener index **(A)**, aboveground biomass of the community **(B)**, aboveground biomass of grasses **(C)**, aboveground biomass of legumes **(D)**, aboveground biomass of sedges **(E)**, and aboveground biomass of forbs **(F)** in an alpine meadow. CK, control; W, warming; P, precipitation increase; WP, the combined warming and precipitation increase. Data are shown as means ± SE. Lowercase letters indicate differences between treatments (Duncan’s multiple comparison post hoc test: P<0.05).

Warming exerted a significant impact on Shannon-Wiener index (*P*<0.01, **Figure 3A**), the aboveground biomass of legumes (*P*<0.01, **Figure 3D**) and sedges (*P*<0.001, **Figure 3E**), but had non-significant influences on the aboveground biomass of the community, grasses and forbs (*P*>0.05, **Figures 3B, C, F**). Precipitation increase had a significant effect on total aboveground biomass of community (*P*<0.01, **Figure 3B**), but did not significantly affect the Shannon-Wiener index (*P*>0.05, **Figure 3A**) and aboveground biomass of four functional groups (*P*>0.05, **Figures 3C–F**).

### Functional groups C:N:P stoichiometry

**Figure 4:**
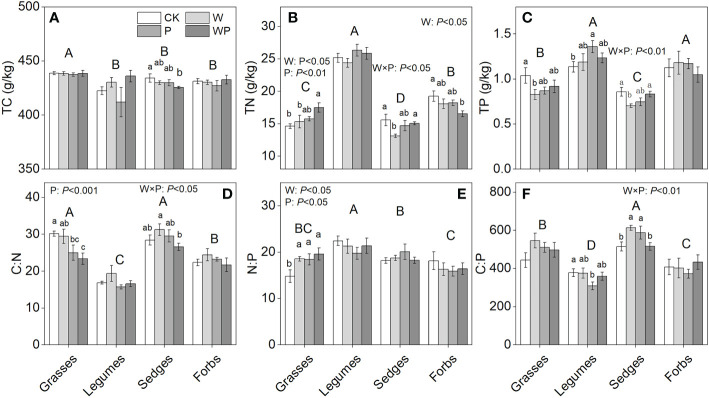
Plant total C (TC), total N (TN), total P (TP) concentration, and C:N:P stoichiometry of four functional plant groups under treatments in an alpine meadow. TC **(A)**, TN **(B)**, TP **(C)**, C:N **(D)**, N:P **(E)**, and C:P **(F)**. CK, control; W, warming; P, precipitation increase; WP, the combined warming and precipitation increase. Data are shown as means ± SE. *P<0.05, **P<0.01. Lowercase letters indicate differences among treatments, and uppercase letters indicate significant differences among four functional groups (Duncan’s multiple comparison post hoc test: P<0.05).

Four functional groups significantly differed in TC, TN, and TP concentrations and C:N:P stoichiometry (**Figure 4**). Warming significantly affected TN concentration in grasses and plant N:P ratios in grasses (*P*<0.05, **Figures 4B, E**). Precipitation increase had significantly positive effects on TN concentration (*P*<0.01, **Figure 4B**) and plant N:P ratio (*P*<0.05, **Figure 4E**) in grasses. The plant C:N ratio in grasses showed a negative response to precipitation increase (*P*<0.001, **Figure 4D**), whereas other functional groups showed non-significant responses. Interaction of warming and precipitation increase had significant effects on TN, TP concentrations, and plant C:N, C:P ratios in sedges (*P*<0.05, **Figures 3B–D, F**). Antagonistic interactions were observed in plant N:P ratio of four functional groups and plant C:P ratio of grasses, legumes, and sedges (**Table 1**).

**Table 1:** The individual effect sizes of warming and precipitation increase, as well as their interaction effect sizes with 95% confidence intervals on the plant total C (TC), total N (TN), total P (TP) concentration and C:N:P stoichiometry of four functional groups.

Response variable		Individual effect sizes of warming	Individual effect sizes of precipitation increase	Warming and precipitation increase interaction effect sizes	Lower 95% C.L.	Upper 95% C.L.	Interaction type
Grasses	TC	0.25	-0.35	0.33	-0.02	0.68	additive
	TN	1.84	2.47	0.77	0.41	1.13	synergistic
	TP	-1.21	-0.59	1.93	1.55	2.30	antagonistic
	C/N	-0.69	-3.31	-0.26	-0.61	0.09	additive
	N/P	2.11	2.00	-1.10	-1.46	-0.74	antagonistic
	C/P	1.21	0.24	-1.56	-1.93	-1.19	antagonistic
Legumes	TC	1.98	-0.28	1.00	0.64	1.36	synergistic
	TN	-0.67	1.43	0.16	-0.19	0.51	additive
	TP	-0.52	1.88	-1.24	-1.61	-0.88	antagonistic
	C/N	1.37	-1.59	-0.67	-1.03	-0.31	synergistic
	N/P	0.17	-0.91	0.97	0.61	1.33	antagonistic
	C/P	1.05	-1.88	1.17	0.81	1.53	antagonistic
Sedges	TC	-1.60	-1.58	-0.01	-0.36	0.34	additive
	TN	-1.65	0.85	2.19	1.81	2.57	antagonistic
	TP	-0.90	0.24	3.15	2.74	3.55	antagonistic
	C/N	-0.04	-1.21	-1.99	-2.37	-1.62	synergistic
	N/P	-0.66	0.68	-1.17	-1.53	-0.81	antagonistic
	C/P	0.52	-0.49	-3.47	-3.88	-3.05	antagonistic
Forbs	TC	0.62	-0.24	0.93	0.57	1.29	synergistic
	TN	-2.26	-2.00	-0.41	-0.77	-0.06	synergistic
	TP	-0.33	-0.44	-0.91	-1.27	-0.56	synergistic
	C/N	0.21	-0.68	-1.32	-1.68	-0.95	synergistic
	N/P	-0.45	-0.73	0.80	0.44	1.16	antagonistic
	C/P	0.68	-0.05	0.83	0.47	1.18	synergistic

### Community C:N:P stoichiometry

**Figure 5:**
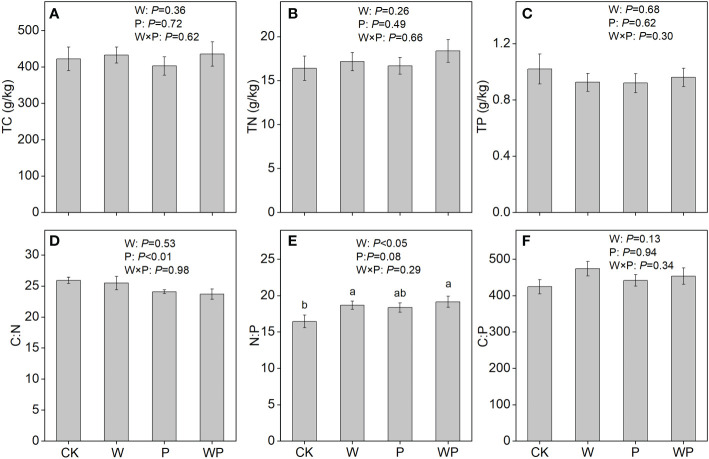
Plant total C (TC), total N (TN), total P (TP) concentration, and C:N:P stoichiometry at community level under treatments in an alpine meadow. TC **(A)**, TN **(B)**, TP **(C)**, C:N **(D)**, N:P **(E)**, and C:P **(F)**. CK, control; W, warming; P, precipitation increase; WP, the combined warming and precipitation increase. Data are shown as means ± SE. Lowercase letters indicate differences between treatments (Duncan’s multiple comparison post hoc test: P<0.05).

**Figure 6:**
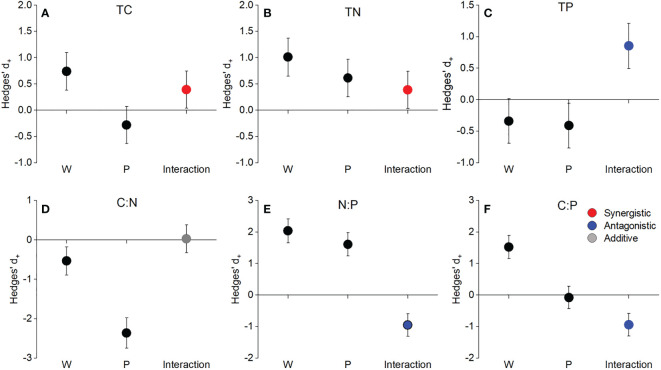
The main and interactive effects of warming and precipitation increase on community total C (TC), total N (TN), total P (TP) concentration, and C:N:P stoichiometry. TC **(A)**, TN **(B)**, TP **(C)**, C:N **(D)**, N:P **(E)**, and C:P **(F)**. Values represent means with 95% confidence intervals (CIs).

Warming and precipitation increase had no significant effects on community TC, TN, TP concentrations, and C:P ratio (**Figures 5A–C, F**). Precipitation increase significantly decreased community C:N ratio (*P*<0.01, **Figure 5D**), whereas warming did not affect plant C:N ratio at community level. Warming had a positive effect on community N:P ratio (*P*<0.05, **Figure 5E**), but precipitation increase did not affect plant N:P ratio at community level. Warming and precipitation increase interactively influenced community TC, TN, TP concentrations, and C:N:P stoichiometry (**Figure 6**). The interactive effects of warming and precipitation increase on soil TC and TN concentrations were synergistic (**Figures 6A, B**). There was an additive interaction in community C:N ratio (**Figure 6D**), while antagonistic interactions were observed in community TP concentration, N:P, and C:P ratios (**Figures 6C, E, F**).

### Factors determining community C:N:P stoichiometry

**Figure 7:**
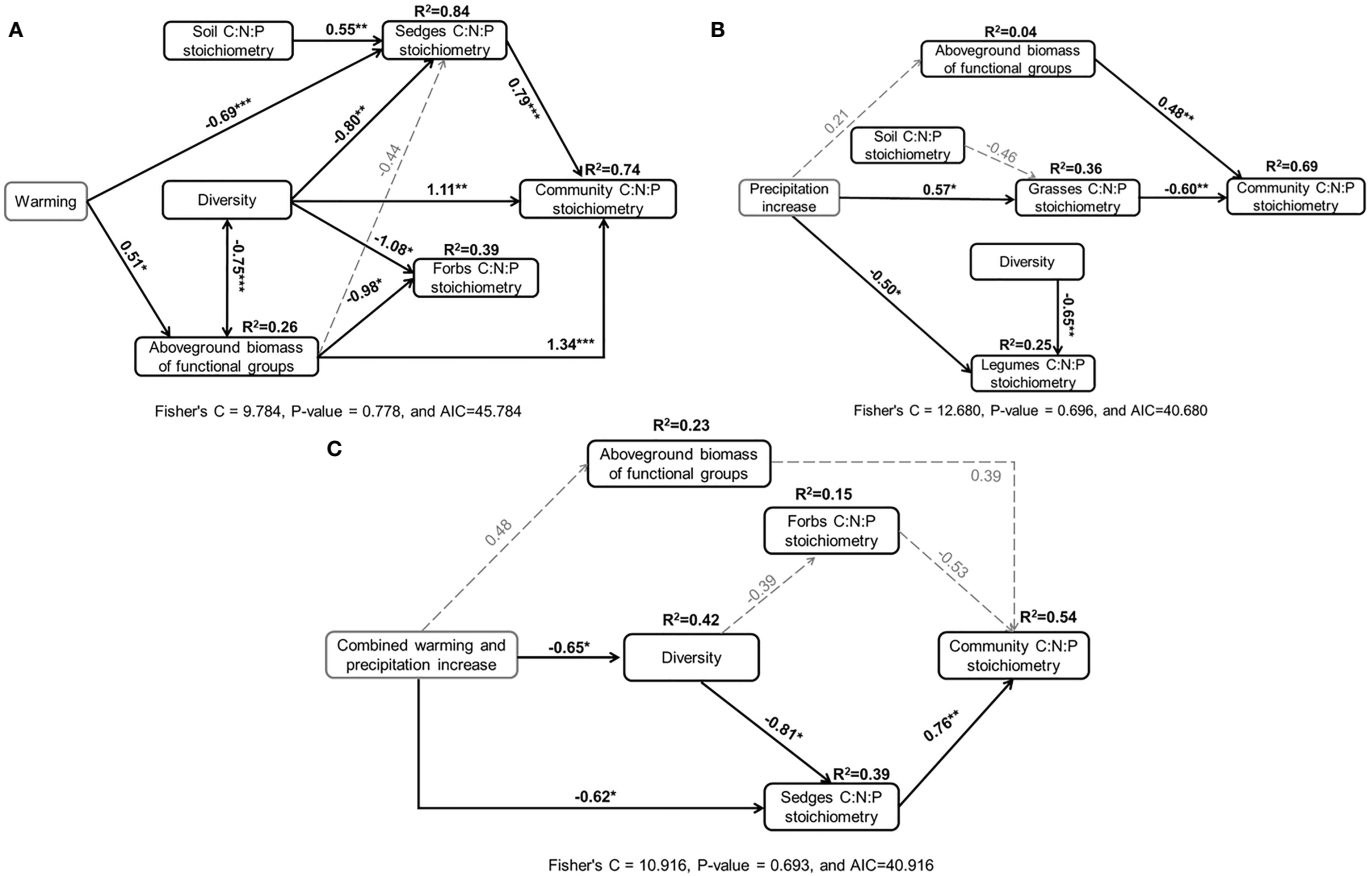
The piecewise structural equation modes performed for the scenarios of warming **(A)**, precipitation increase **(B)**, and combined warming and precipitation **(C)**, linking community C:N:P stoichiometry to soil and plant characteristics. Solid arrows indicate significantly directions and effects (*P<0.05, **P<0.01, ***P<0.001), and grey dashed arrows denote non-significant directions and effects but crucial to the final model fit (P>0.05). Numbers associated with solid and dashed arrows indicated the standard path coefficient. R2 indicates the variance explained by the models of each dependent variable.

Based on our prior conceptual models (**Figure S3**), the final SEMs predicted that warming, precipitation increase, and their combination had significant effects on community C:N:P stoichiometry (**Figure 7**). The SEMs indicated all predictor variables included in the models explained 74%, 69%, and 54% of the variance in community C:N:P stoichiometry in warming, precipitation increase, and their combination plots, respectively. The positive and direct effects on community C:N:P stoichiometry were primarily due to changes in the sedges C:N:P stoichiometry, diversity, and aboveground biomass of functional groups in the warming plot (**Figure 7A**). Precipitation increase indirectly affected community C:N:P stoichiometry through variation in grasses C:N:P stoichiometry (**Figure 7B**). The significant indirect effects of combined warming and precipitation increase on community C:N:P stoichiometry were mainly due to change in sedges C:N:P stoichiometry (**Figure 7C**).

## Discussion

Soil nutrient concentration and C:N:P stoichiometry reflect C cycle, soil fertility, and available nutrient in the terrestrial ecosystems (Whitehead et al., 2018; Li et al., 2021a). Plant C:N and C:P ratios imply the N and P use efficiency, respectively (Patterson et al., 1997), and N:P ratio reflects the nutrient limit during plant growth (Güsewell, 2004). Our results showed that warming and precipitation increase showed antagonistic effects on plant and soil N:P and C:P ratios, indicating that precipitation increase offsets the effect of warming on N:P and C:P ratios of plant and soil. Our results provided evidence for the individual, combined, and interactive effects of warming and precipitation increase on plant and soil C:N:P stoichiometry in an alpine meadow.

### Effects of warming on plant and soil C:N:P stoichiometry

Conflicted with our first hypothesis, warming did not alter soil TC, TN, TP concentrations and C:N:P stoichiometry (**Figure 1**), similar observations have been made in the previous studies that the effects of warming on soil nutrition and C:N:P stoichiometry appear to be negligible (Wang et al., 2014; Yu et al., 2019a). These consistent results indicate that soil has high stoichiometric homeostasis under future warming scenarios in an alpine meadow, which is supported by previous meta-analysis (Yue et al., 2017) and case study (Xu et al., 2022).

Warming exerted a significantly negative impact on plant diversity, which is consistent with previous studies from the Tibetan plateau (Ganjurjav et al., 2016) and the Arctic tundra (Alatalo et al., 2021). This phenomenon might be explained by the opposite effect of warming on legumes and sedges. In this study, warming significantly increased the aboveground biomass of legumes (**Figure 3D**), which is in line with previous studies in alpine meadows (Wang et al., 2012; Xu et al., 2018). On the contrary, the aboveground biomass of sedges was significantly decreased by warming (**Figure 3E**). The result is explicable by the fact that OTCs warming promotes soil evaporation and plant evapotranspiration, resulting in a reduction of soil moisture, which is not conducive to the growth of sedges with roots distributed in the shallow soil (Ganjurjav et al., 2016; Xu et al., 2018). Warming did not influence the aboveground of grasses and forbs in our experiment (**Figures 3C, F**), which implies that these two functional groups were less sensitive to warming.

The C:N:P stoichiometry of four functional groups had distinct responses to warming (**Figure 4**). These results supported our first hypothesis that warming significantly change plant C:N:P stoichiometry. Given the significantly increased TN concentration and unchanged TP concentration in response to warming, a significantly positive response of N:P ratio was observed in grasses. Furthermore, the N:P ratio of grasses under the warming treatment was 18.57, which was significantly higher than that of 14.81 under the CK. The uncoupled change in TN and TP concentration of grasses indicated warming shifted grasses from N and P limitation to P limitation (Koerselman and Arthur, 1996; Güsewell, 2004).

The community C:N:P stoichiometry of ecosystem reflects the sum impacts of the functional groups (Bai et al., 2012; Ning et al., 2021). Hence, the community-level C:N:P stoichiometry is a strong indication of the elemental balance of the ecosystems (Ning et al., 2021). Our results showed that warming tended to increase community N:P ratio (**Figure 5E**), which is comparable with similar findings in previous study (Yue et al., 2017; Zhou et al., 2021). These results may be attributable primarily to enhanced P dilution of plant (Zong et al., 2018), and facilitated N uptake of plant caused by the increased rates of soil N mineralization and nitrification under warming (Melillo et al., 2011). This may be an indication that warming alleviates N limitation and increase P limitation of plant growth (N:P ratio=18.67) (Dijkstra et al., 2012). However, the enhanced plant N:P ratio induced by warming was inconsistent with those results obtained from typical and meadow steppes (Yan et al., 2022). These distinct observations might be attributed to difference in the limited environmental factors. Moreover, SEM indicated that the aboveground biomass of functional groups played a more crucial role than sedges C:N:P stoichiometry and diversity in regulating the response of community C:N:P stoichiometry to warming (**Figure 7A**). Given that grasses were the functional group with the highest relative biomass, the positive response of their N:P ratio resulted in a significant higher community N:P ratio under warming. Although warming had a negative effect on total N concentration of forbs, the minor contribution of forbs to community biomass (< 15%) resulted in a non-significant effect on community total N concentration.

### Effects of precipitation increase on plant and soil C:N:P stoichiometry

Our results indicated that soil nutrients had no significant responses to precipitation increase (**Figures 1A–C**), suggesting that increased 20% rainfall hardly affects soil fertility. The negative response of soil C:N ratio to precipitation increase is probably because the increased rainfall alleviates N-limitation (Yuan and Chen, 2015; Li et al., 2021b). The result supports our first hypothesis that soil C:N:P stoichiometry respond to precipitation increase. The higher N:P and C:P ratios under precipitation increase compared with control were partially owing to marginally increased soil TP concentration.

The availability of water, nutrients, and other resources shape plant community composition (Tilman, 1987). In line with previous studies (Ponce-Campos et al., 2013; Xu et al., 2018), our results showed that precipitation increase significantly increased total aboveground biomass (**Figure 3B**). The positive response of total aboveground biomass might attribute to a remarkable increase in aboveground biomass of grasses. TN concentration of grasses had a positive response to precipitation increase (**Figure 4B**). A possible mechanism for the result is that grasses often become increasingly dominant under water supply due to their higher ability to absorb nutrients, whereas other functional groups are located on the relatively low canopy, so it has weak light competition compared with the upper grasses (Collins et al., 2012; Li et al., 2019). Although precipitation increase did not significantly change TC concentration of grasses, the C:N ratio was decreased significantly due to the significant increase in TN concentration of grasses. In addition, the positive response of N:P ratio in grasses to precipitation increase indicated that the growth of grasses might be limited by P concentration. It was noteworthy that legumes had a lower C:P ratio under precipitation increase relative to control due to unchanged TC concentration and increased TP concentration. This may be an indication that increased rainfall facilitates the uptake of P by legumes (Zhang et al., 2020b).

There were no significant responses of plant TC, TN, and TP concentrations to precipitation increase (**Figures 5A–C**). Given that grasses were the functional group with the highest relative biomass, the negative response of their C:N ratio resulted in a significantly negative effect of precipitation increase on community C:N ratio. This result was also verified in SEM that grasses C:N:P stoichiometry and aboveground biomass of functional groups mediated the impact of precipitation increase on community C:N:P stoichiometry (**Figure 7B**). Lower C:N ratio at community level indicated that precipitation increase inhibited the plant N use efficiency and promote plant growth to compete for resources such as water in favorable environments (Zhang et al., 2020a). Non-significant N:P and C:P ratios indicated that precipitation increase did not alter nutrient limitation and P use efficiency during plant growth.

### Antagonistic effects of warming and precipitation increase on plant and soil C:N:P stoichiometry

Actually, global climate change usually involves the simultaneous occurrence of multiple climate factors (e.g., warming and altered precipitation), which interactively affect plant and soil C:N:P stoichiometry (Yuan and Chen, 2015; Yue et al., 2017). Confirming our second hypothesis, antagonistic effects dominated the response of soil total P concentration, C:N, N:P, and C:P ratios to warming and precipitation increase (**Figures 2C–F**). Furthermore, warming greatly dampened the effects of precipitation increase on soil N:P and C:P ratios, resulting in non-significant effects of the combined treatment. These results indicated that the interactive effect of warming and precipitation increase on soil C:N:P stoichiometry was weaker than that of single factor treatment (Larsen et al., 2011; Leuzinger et al., 2011). This is because precipitation increase mitigates or even offsets the inhibition of soil water availability caused by warming (Yu et al., 2019b; Zhao et al., 2019).

The response of functional groups to the combination and interaction of warming and precipitation increase was inconsistent from the perspective of plant C:N:P stoichiometry (**Figures 4**, **5**). Precipitation increase dampened the effects of warming on total N and P concentrations and C:P ratio of sedges, resulting in non-significant effects of the combined treatment. The significant effect of the interaction of warming and precipitation on TN and TP concentrations and non-significant TC concentration in sedges caused significant responses of C:N and C:P ratios and non-significant response of N:P ratio. Warming and precipitation increase had antagonistic effects on N:P and C:P ratios of grasses, legumes, and sedges, thus exacerbating nutrient limitation and inhibiting P use efficiency (Yan et al., 2022).

At the community level, precipitation increase weakly suppressed the positive effect of warming on plant N:P ratio due to their antagonistic effects. As a result, precipitation increase alleviated P limitation for plant growth caused by warming. However, the combined treatment of warming and precipitation increase still had a higher community N:P ratio relative to the control, indicating that the inhibited effect of precipitation increase on the warming-induced increase in plant N:P ratio was non-significant.

## Conclusion

Warming and precipitation increase affected soil and plant C:N:P stoichiometry to some extent. First, soil C:N:P stoichiometry showed high stoichiometric homeostasis to warming and the interaction of warming and precipitation, whereas precipitation increase had a significantly negative effect on soil C:N ratio. The combined treatment had no significant effects on soil N:P and C:P ratios due to antagonistic interaction between warming and precipitation increase. Second, warming shifted plant community structure to more legumes and fewer sedges. Warming increased grasses and community N:P ratios, indicating the shift in plant nutrient limitation from N and P limitation to P limitation. Precipitation increase enhanced the productivity of alpine meadows, and reduced C:N ratio of grasses and community. Finally, warming and precipitation increase had antagonistic effects on N:P and C:P ratios of soil and plant. Although precipitation increase alleviated warming-induced P limitation of plant growth, the combined treatment of warming and precipitation increase still had a higher community N:P ratio relative to the control. This study demonstrated that warming and precipitation increase could interact antagonistically on N:P and C:P ratios of plant and soil in alpine meadows, which might have important implications for predicting the future nutrient cycle of high-altitude grassland ecosystems. Therefore, the interactive effects between warming and precipitation increase should be taken into account when assessing the effects of climate change on biogeochemical cycles in grassland ecosystems.

## Data availability statement

The original contributions presented in the study are included in the article **Supplementary Material**, further inquiries can be directed to the corresponding author.

## Author contributions

XS and KL conceived the study, supervised the writing, and revised the manuscript. LS led the writing. ZL, XW, CP, ZY, BH, and QX contributed sections to the manuscript. HZ and XS provided funding support. All authors read and approved the final submission.

## Funding

This work was financially supported by the National Natural Science Foundation of China (Grant No. 32171685 and 31971746), the second batch of forestry and grassland ecological protection and restoration funds in 2020: Qilian Mountain National Park Qinghai Area Biodiversity Conservation Project (QHTX-2021-009), Qinghai Province Innovation Platform Construction Special Project (2022-ZJ-Y02), and National Natural Science Foundation of China Joint Fund Project (U21A20186).

## Conflict of interest

The authors declare that the research was conducted in the absence of any commercial or financial relationships that could be construed as a potential conflict of interest.

## Publisher’s note

All claims expressed in this article are solely those of the authors and do not necessarily represent those of their affiliated organizations, or those of the publisher, the editors and the reviewers. Any product that may be evaluated in this article, or claim that may be made by its manufacturer, is not guaranteed or endorsed by the publisher.
