# Supplementary material for: Precipitation increase counteracts warming effects on plant and soil C:N:P stoichiometry in an alpine meadow
Source: Front Plant Sci. 2022 Nov 2;13:1044173. doi: 10.3389/fpls.2022.1044173 (PMC9666903; doi:10.3389/fpls.2022.1044173)
Supplement: Supplementary file 1 [file DataSheet_1.docx]

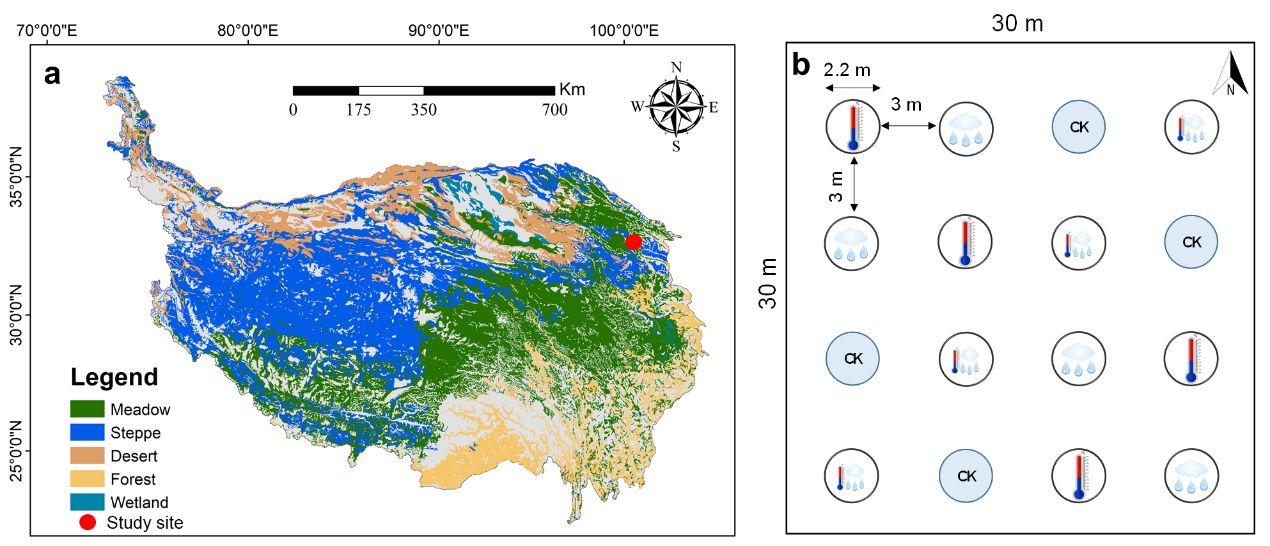


**Fig. S1.** Study site at the alpine meadow (a) and the schematic diagram of experimental design (b).


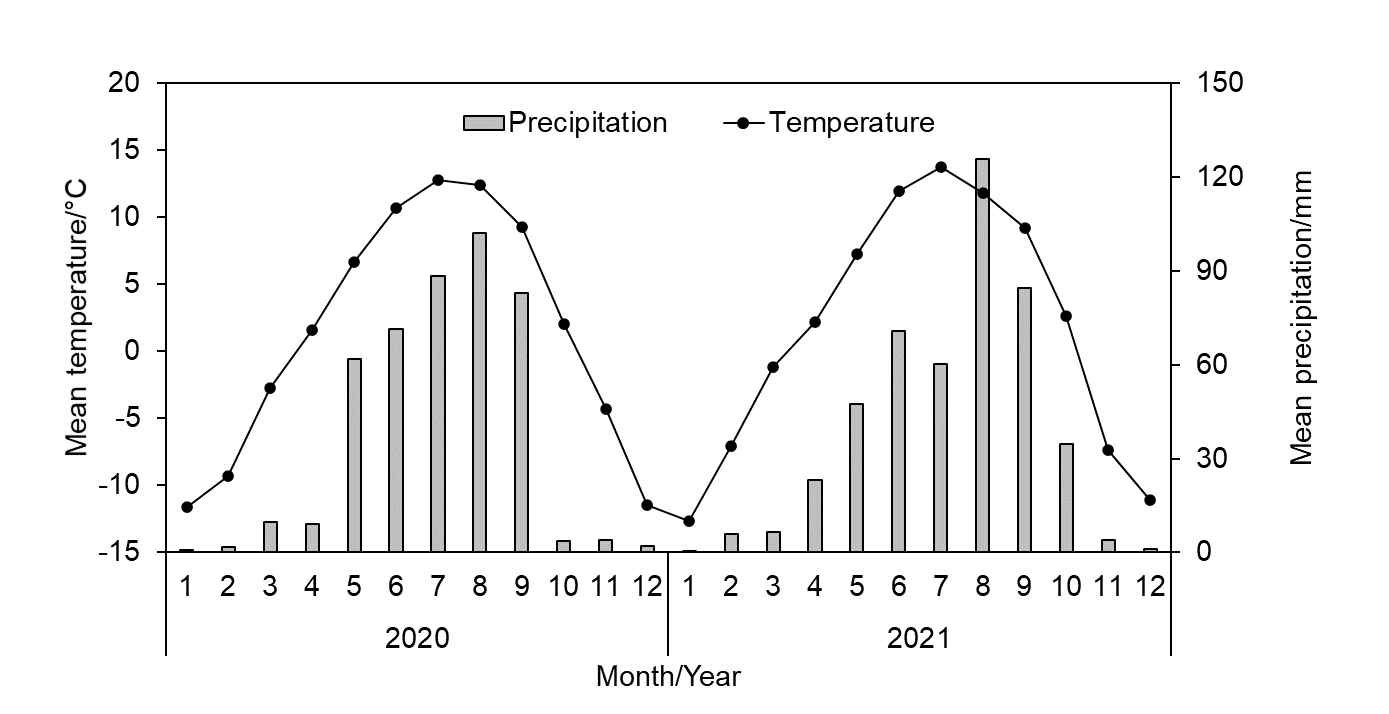


**Fig. S2.** Mean monthly temperature and precipitation of the study site from 2020 to 2021.


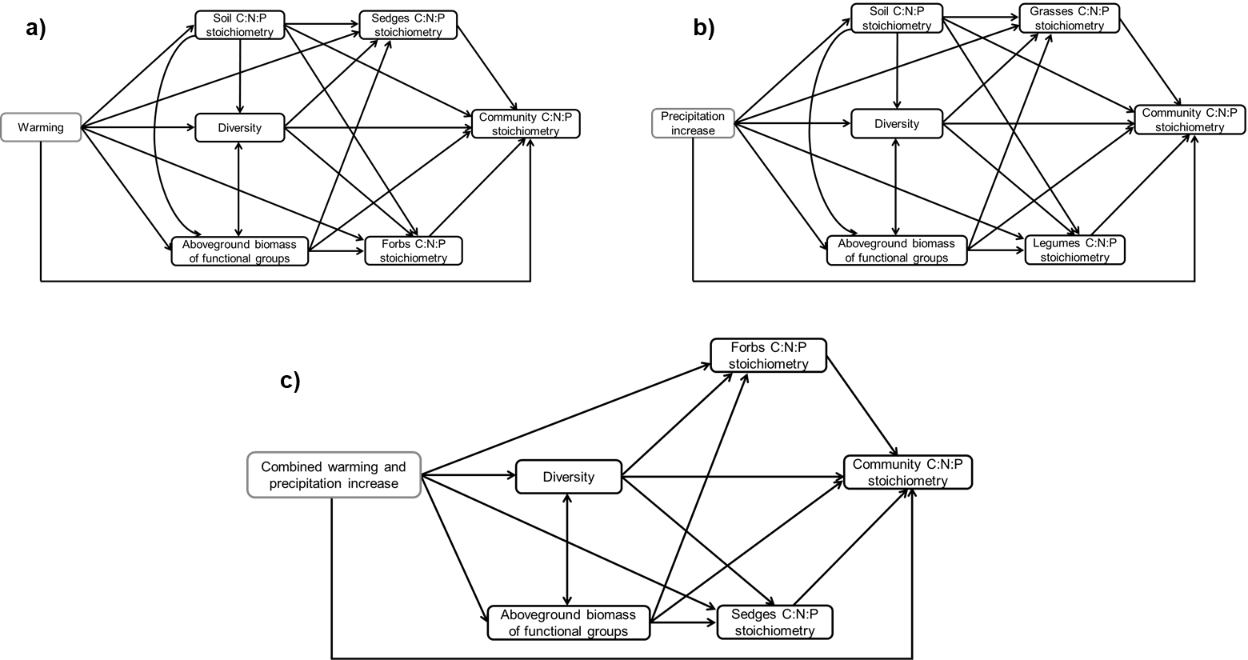


**Fig. S3.** Hypothetical causal model for structural equation modelling (SEM) exploring the effects of warming (a), precipitation increase (b), and combined warming and precipitation increase (c), linking community C:N:P stoichiometry to soil and plant characteristics. Black arrows are hypothesized paths.
